# Enhanced Light Emission due to Formation of Semi-polar InGaN/GaN Multi-quantum Wells

**DOI:** 10.1186/s11671-015-1171-1

**Published:** 2015-12-01

**Authors:** Wan-Ru Zhao, Guo-En Weng, Jian-Yu Wang, Jiang-Yong Zhang, Hong-Wei Liang, Takashi Sekiguchi, Bao-Ping Zhang

**Affiliations:** Department of Electronic Engineering, Xiamen University, Xiamen, 361005 People’s Republic of China; Department of Physics, Xiamen University, Xiamen, 361005 People’s Republic of China; School of Electronic Science and Engineering, Nanjing University, Nanjing, 210093 People’s Republic of China; School of Physics and Optoelectronic Engineering, Dalian University of Technology, Dalian, 116024 People’s Republic of China; World Premier International (WPI) Center for Materials Nanoarchitectonics (MANA), National Institute for Materials Science (NIMS), Namiki 1-1, Tsukuba, Ibaraki 305-0044 Japan

**Keywords:** Semi-polar, InGaN/GaN multi-quantum wells, Cathodoluminescence, Photoluminescence

## Abstract

InGaN/GaN multi-quantum wells (MQWs) are grown on (0001) sapphire substrates by metal organic chemical vapor deposition (MOCVD) with special growth parameters to form V-shaped pits simultaneously. Measurements by atomic force microscopy (AFM) and transmission electron microscopy (TEM) demonstrate the formation of MQWs on both (0001) and ($1\overline{1}01$) side surface of the V-shaped pits. The latter is known to be a semi-polar surface. Optical characterizations together with theoretical calculation enable us to identify the optical transitions from these MQWs. The layer thickness on ($1\overline{1}01$) surface is smaller than that on (0001) surface, and the energy level in the ($1\overline{1}01$) semi-polar quantum well (QW) is higher than in the (0001) QW. As the sample temperature is increased from 15 K, the integrated cathodoluminescence (CL) intensity of (0001) MQWs increases first and then decreases while that of the ($1\overline{1}01$) MQWs decreases monotonically. The integrated photoluminescence (PL) intensity of (0001) MQWs increases significantly from 15 to 70 K. These results are explained by carrier injection from ($1\overline{1}01$) to (0001) MQWs due to thermal excitation. It is therefore concluded that the emission efficiency of (0001) MQWs at high temperatures can be greatly improved due to the formation of semi-polar MQWs.

## Background

Recently, GaN based light-emitting diodes (LEDs) with InGaN/GaN multi-quantum wells (MQWs) as the active region is being widely used in the field of solid-state semiconductor lighting (SSL). However, typical InGaN/GaN MQW LEDs grown on (0001) sapphire substrate suffer from high threading dislocations density (10^8^/cm^2^–10^10^/cm^2^) caused by mismatch in lattice constants and in thermal expansion coefficients between the GaN-based materials and the sapphire substrate [1, 2] and strong quantum-confined Stark effect (QCSE) due to the large internal electric field originated from both spontaneous polarization, induced by wurtzite crystal structure of GaN materials, and piezoelectric polarization induced by strain effect [3–6]. They both can cause a heavy “efficiency droop” [7]. To overcome these problems, there are many methods have been reported to enhance the light emission efficiency, such as using GaN substrate [8] and lateral epitaxial overgrowth [9]. These methods, however, cannot be widely used because of the high cost. Consequently, semi-polar ($1\overline{1}01$) quantum well (QW) grown in the sidewall of the V-shaped pits is attracting attentions [10–12] because, unlike conventional (0001) QW, it has very weak QCSE due to very small internal electric field. Additionally, the In incorporation efficiency has been proposed to be higher in semi-polar InGaN/GaN MQWs than in polar InGaN/GaN MQWs [13]. Therefore, the semi-polar InGaN/GaN MQWs are beneficial for fabricating high-efficiency GaN-based optoelectronic devices.

The promising application of semi-polar InGaN/GaN MQWs has inspired more and more research teams to study its emission properties. It has been reported that growing semi-polar InGaN/GaN MQWs in V-shaped pits could increase the light emission efficiency by A. Hangleiter et al. [10] and S. H. Han et al. [11]. The reason has been ascribed to an energy barrier around the V-shaped pit which keeps carriers from reaching the dislocation and recombining nonradiatively. In this paper, we also report an improvement of light emission efficiency in the (0001) InGaN/GaN MQWs sample with forming semi-polar ($1\overline{1}01$) InGaN/GaN MQWs in the sidewall of the V-shaped pits. An important phenomenon is observed here to explain why the light emission efficiency of the sample could be enhanced through forming semi-polar InGaN/GaN MQWs, which is proposed for the first time.

## Methods

**Fig. 1:**
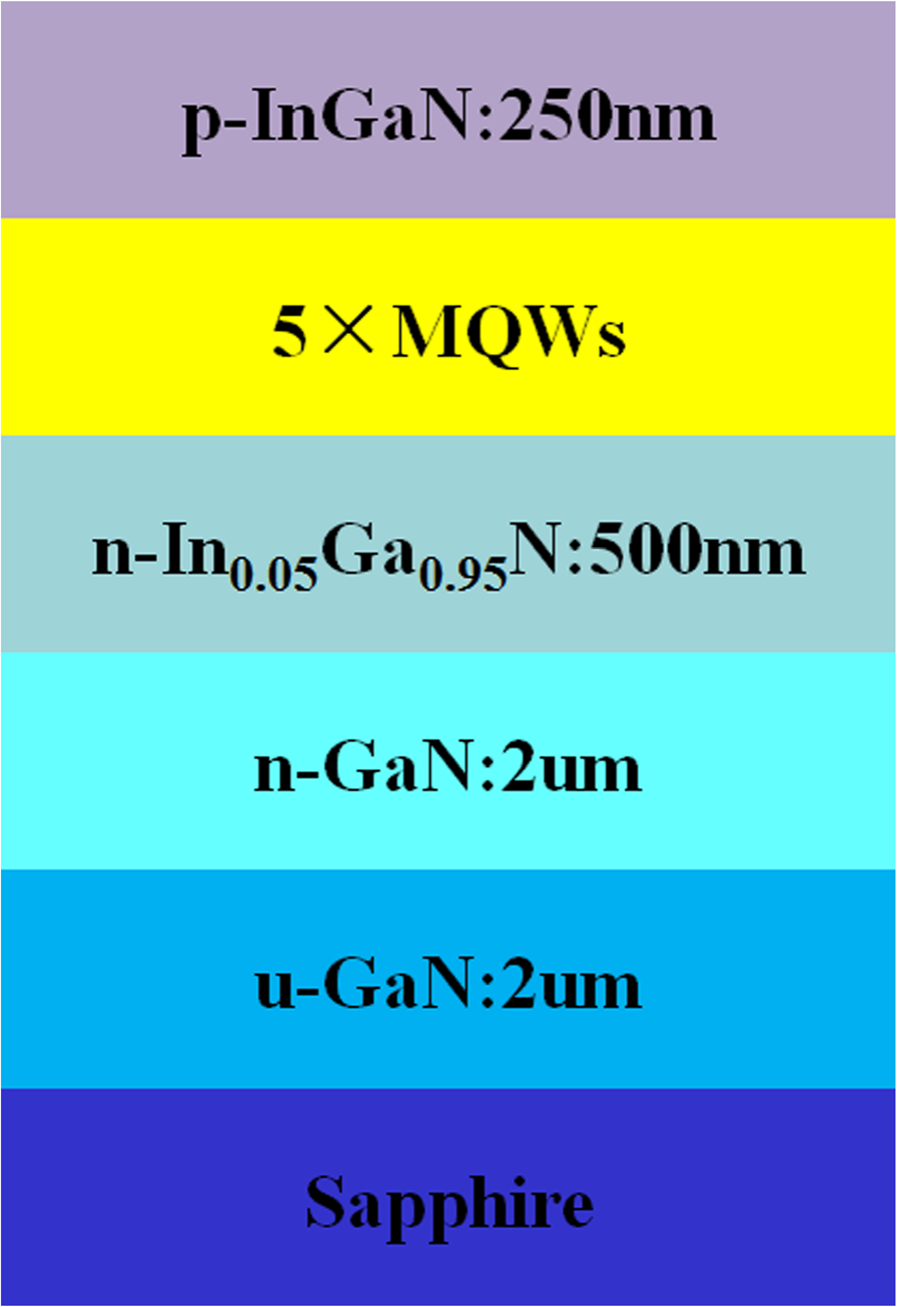
Schematic illustration of the sample

The InGaN/GaN MQW sample was epitaxially grown on a (0001)-oriented sapphire substrate by metal organic chemical vapor deposition (MOCVD) with forming a lot of V-shaped pits simultaneously under special growth conditions. Trimethylgallium (TMGa), trimethylindium (TMIn), and ammonia (NH_3_) were used as precursors for Ga, In, and N, while silane (SiH_4_) and biscyclopentadienyl magnesium (CP_2_Mg) were used as n-type and p-type dopants, respectively. The epitaxial structure includes 2 μm un-doped-GaN layer, 2 μm n-GaN layer, 500 nm n-In_0.05_Ga_0.95_N layer, five-pairs In_0.23_Ga_0.77_N/GaN MQWs containing polar InGaN/GaN MQWs in (0001) plane and semi-polar InGaN/GaN MQWs in ($1\overline{1}01$) plane of the V-shaped pits, and 250 nm p-InGaN layer. The epitaxial structure of the sample is shown in Fig. 1.

The sample’s microstructure properties are measured by atomic force microscopy (AFM) and transmission electron microscopy (TEM). The electron and hole quantized energy levels and wave functions of the two kinds of QWs are calculated by using finite difference method. Temperature-dependent cathodoluminescence (CL) and photoluminescence (PL) experiments are used to measure the sample’s light emission properties. The CL experiments are performed with the incident 5-keV high-energy electron beam focused on the V-shaped pit over a temperature range from 15 to 200 K. The luminescence signal is collected by a parabolic mirror, then dispersed by a monochromator, and detected by a charge-coupled device (CCD). The PL measurements were performed with the samples held in a helium closed-circuit refrigerator over a temperature range from 15 to 300 K. The PL signal is also dispersed by a monochromator and detected by a CDD. A 405-nm continuous wave (CW) semiconductor laser is used as the PL excitation source and large-area excitation.

## Results and Discussion

**Fig. 2:**
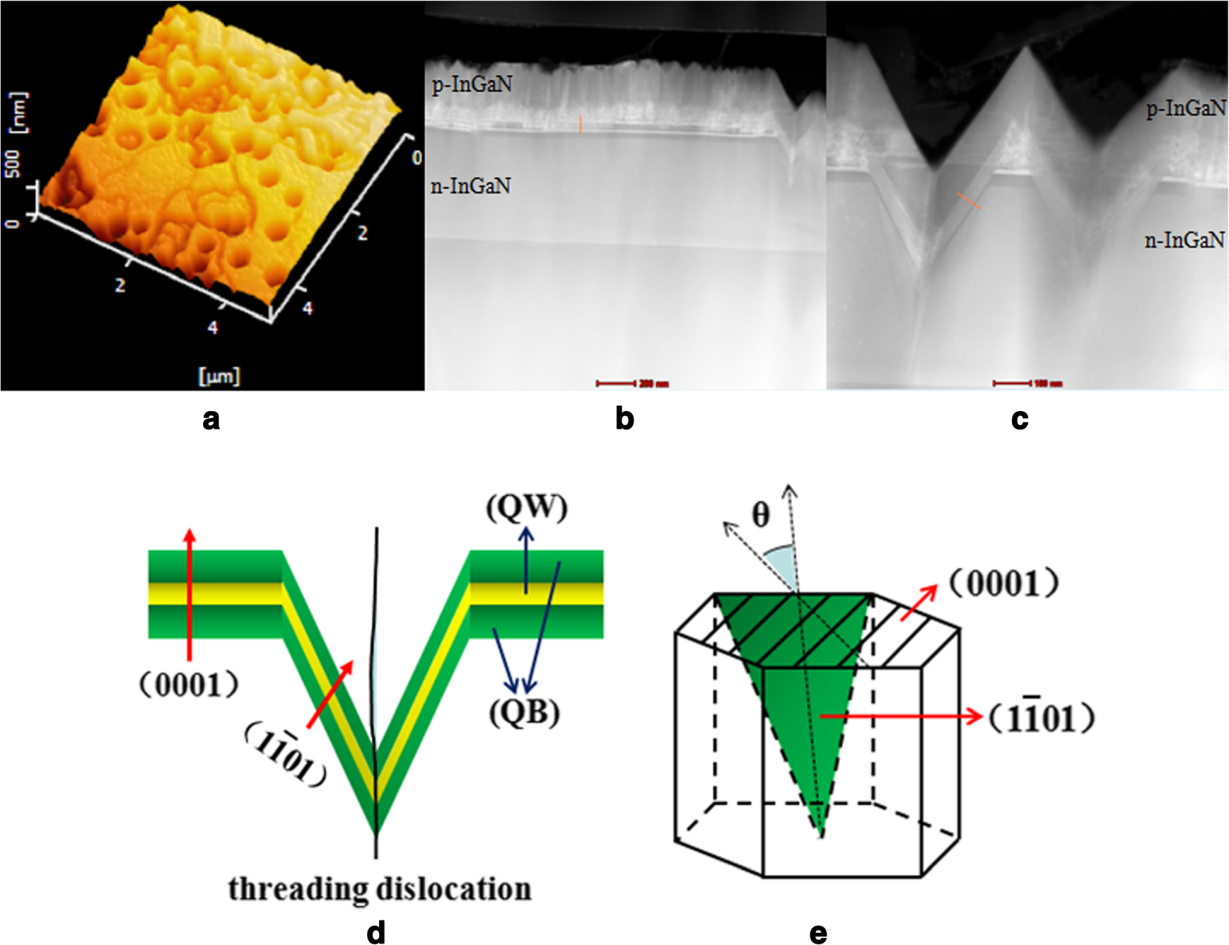
**a** 5 μm×5 μm AFM image. **b** Cross-sectional TEM of (0001) area. **c** TEM image of V-shaped pit. **d** Schematic illustration of a period semi-polar QW and (0001) QW. **e** Schematic illustration of (0001) plane and (1$\overline{1}$01) plane

Figure 2a shows the 5 μm × 5 μm AFM image of the surface of the sample; a lot of V-shaped pits are clearly observed. Figure 2b shows the cross-sectional TEM image of the (0001) area. Figure 2c shows the TEM image of the V-shaped pits. A threading dislocation exists in the inside of the V-shaped pit. The QW grown on (0001) plane has a thickness of 4 nm and a period of 17 nm. However, the semi-polar QW in the V-shaped pits grown on ($1\overline{1}01$) plane exhibits a much thinner thickness than that of c-plane QW which has a thickness of 1.6 nm and a period of 6.9 nm. It is because that the atomic adhesion is different between the (0001) plane and ($1\overline{1}01$) plane, which leading to the faster growth rate on (0001) surface than on ($1\overline{1}01$) surface. The structure of a period semi-polar QW and (0001) QW is also shown schematically in Fig. 2d, e. The angle between (0001) plane and ($1\overline{1}01$) plane is *θ*.

We here calculate the electron and hole quantized energy levels and wave functions of the two kinds of QWs by using finite difference method based on the Schrodinger’s equation:

1 $$ -\frac{\hslash^2}{2{m}^{*}}\cdot \frac{d^2}{d{x}^2}\psi (x)+V(x)\cdot \psi (x)={E}_n\cdot \psi (x) $$

where *ћ* is the reduced Planck constant, m* is the electron or hole effective mass, *V*(*x*) is the energy band, *ψ*(*x*) is the wave function, *E*_*n*_ is the energy level. To calculate *ψ*(*x*) and *E*_*n*_, we must know *V*(*x*). And, *V*(*x*) is influenced by electrostatic field in QW, so we should calculate the electrostatic field first. For the periodic MQW structure, the change values of electric potential of well layer and barrier layer in each period are the same, just opposite in sign. The electrostatic field of one period QW grown by materials of thickness *L* and dielectric constants *ε* can be expressed as [14, 15]:

2 $$ \begin{array}{l}{E}_b=\frac{L_w\left({P}_w-{P}_b\right)}{\varepsilon_0\left({\varepsilon}_w{L}_b+{\varepsilon}_b{L}_w\right)}\\ {}\end{array} $$

3 $$ {E}_w=\frac{L_b\left({P}_b-{P}_w\right)}{\varepsilon_0\left({\varepsilon}_w{L}_b+{\varepsilon}_b{L}_w\right)} $$

where the subscripts *w* and *b* represent the well layer and the barrier layer, respectively. *ε*_0_ is the vacuum dielectric constant, and *P* is the total polarization including spontaneous and piezoelectric. The spontaneous polarization *P*_SP_ and piezoelectric polarization *P*_PZ_ can be calculated by [16–18]:

4 $$ {P}_{\mathrm{SP}}^{(0001)}\left({\mathrm{In}}_x{\mathrm{Ga}}_{1-x}N\right)=-0.042x-0.034\left(1-x\right)+0.037x\left(1-x\right) $$

5 $$ {P}_{\mathrm{PZ}}^{(0001)}\left({\mathrm{In}}_x{\mathrm{Ga}}_{1-x}N\right)=0.148x-0.0424\left(1-x\right) $$

6 $$ {P}_{\mathrm{SP}}^{\left(1\overline{1}01\right)}={P}_{\mathrm{SP}}^{(0001)}\cdot \cos \theta $$

7 $$ \begin{array}{l}{P}_{\mathrm{PZ}}^{\left(1\overline{1}01\right)}={e}_{31}\cdot \cos \theta \cdot {\varepsilon}_{x^{,}{x}^{,}}+\left({e}_{31}\cdot { \cos}^3\theta +\frac{e_{33}-{e}_{15}}{2}\cdot \sin \theta \cdot \sin 2\theta \right)\cdot {\varepsilon}_{y^{,}{y}^{,}}\\ {}+\left(\frac{e_{33}+{e}_{15}}{2}\cdot \sin \theta \cdot \sin 2\theta +{e}_{33}\cdot { \cos}^3\theta \right)\cdot {\varepsilon}_{z^{,}{z}^{,}}\\ {}+\left[\left({e}_{31}-{e}_{33}\right)\cdot \cos \theta \cdot \sin 2\theta +{e}_{15}\cdot \sin \theta \cdot \cos 2\theta \right]\cdot {\varepsilon}_{y^{,}{z}^{,}}\end{array} $$

where *e*_*31*_, *e*_*33*_, and *e*_*15*_ are piezoelectric tensor, *ε*_*x*’*x*’_ , *ε*_*y*’*y*’_ , *ε*_*z*’*z*’_ , and *ε*_*y*’*z*’_ are elastic strain, and *x* is the In content. The material parameters of InGaN are obtained from Vegard’s law [17] by using the parameters of wurtzite GaN and InN which are listed in Table 1. The ratio of conduction band discontinuity to valence band discontinuity is assumed to be 7:3 at the interfaces of InGaN/GaN.

**Table 1:** Material parameters of GaN and InN

	*E*_*g*_	*ε*	*e*_33_	*e*_31_	*e*_15_	*m* e*	*m* h*
	(eV, 300 K)		(C/m^2^)	(C/m^2^)	(C/m^2^)	(*m*_0_)	(*m*_0_)
GaN	3.4	10.28	0.73	−0.49	−0.40	0.20	1.60
InN	0.7	14.61	0.73	−0.49	−0.40	0.11	1.63

**Fig. 3:**
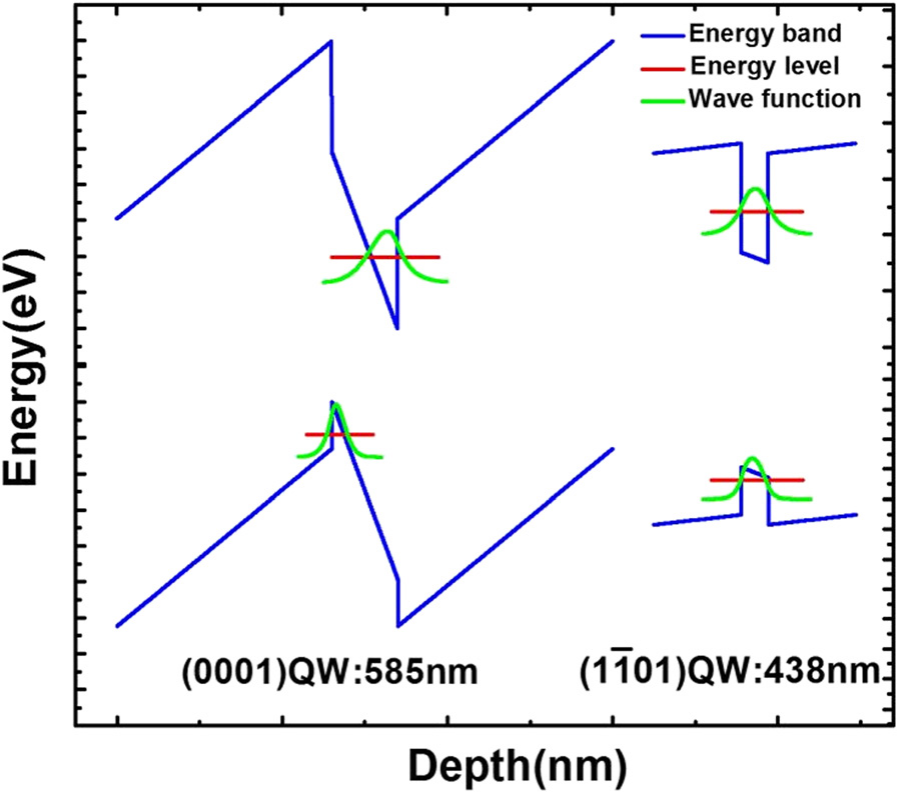
Calculated electron and hole quantized energy levels and wave functions together with energy band structure

Figure 3 shows the calculated electron and hole quantized energy levels and wave functions together with the energy band structure. The light-emitting wavelength of (0001) QW and ($1\overline{1}01$) semi-polar QW is calculated to be 585 and 438 nm, respectively. The emitting photon energy of ($1\overline{1}01$) QW is 355.8 meV higher than that of (0001) QW. This energy barrier is much larger than the thermal excitation energy caused by increasing temperature. So, the carriers in (0001) QW cannot nonradiatively [10, 11]. At the same time, we suspect that the carriers in semi-polar QW are easy to transfer into (0001) QW to recombine and shine. We also observe that the energy band of (0001) QW is more tilted than that of ($1\overline{1}01$) QW. It is because the polarization intensity of (0001) QW is much larger than ($1\overline{1}01$) QW’s. So, the QCSE of (0001) QW is very strong, while that of ($1\overline{1}01$) QW is very weak.

**Fig. 4:**
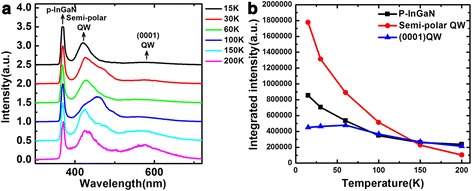
**a** CL spectra of the sample from 15 to 200 K. **b** Dependence of CL integrated intensity on temperature. The electron beam was focused on one V-shaped pit

The CL spectra of the sample at different temperatures from 15 to 200 K are shown in Fig. 4a. It can be seen that the CL spectra contain three light-emitting peaks, 380, 440, and 585 nm. The peak of 380 nm is emitted by the p-InGaN, which is the surface of the sample. The peaks of 440 and 585 nm are emitted by the ($1\overline{1}01$) and (0001) QW, respectively. They are in accordance with the calculated values. In CL measurement, the incident high-energy electron beam is just focused on the V-shaped pit, so the (0001) QW should not be excited in principle. However, we indeed observe the luminescence of (0001) QW from the CL spectra. At the same time, we also find that the peak of 585 nm emitted by (0001) QW is very weak and even could be negligible at the low temperature of 15 K. As the temperature increase, however, the ratio of the intensity of 585-nm peak to the intensity of 440-nm peak increases gradually. It is known that, due to In composition fluctuation, there are lots of localization centers in a common InGaN QW [19]. With increasing temperature, the localized carriers will be thermally activated to escape from the localization centers and become free in the well layer. This is considered to be true even for ($1\overline{1}01$) QW. At higher temperatures, the carrier localized in the localization centers gains enough energy to escape and become free within the ($1\overline{1}01$) QW, which causes increase of carriers transporting from ($1\overline{1}01$) QW to (0001) QW.

The integrated CL intensity versus temperature is plotted in Fig. 4b. The black curve is the CL integrated intensity of p-InGaN. It decreases with the temperature rising due to the capture of carriers by threading dislocation. The red curve is the CL integrated intensity of semi-polar QW. It decreases much significantly than that of p-InGaN. This is because that the carriers in semi-polar QW not only can be captured by threading dislocation but also can transfer into (0001) QW. The CL integrated intensity of (0001) QW is plotted by the blue curve. It first increases and then decreases. The increase is due to transferring of more carriers from semi-polar QW into (0001) QW with elevating temperature. At even higher temperatures, however, the nonradiative recombination [20] gradually plays a leading role, resulting in the followed decrease of the CL integrated intensity of (0001) QW. This confirms our speculation again.

**Fig. 5:**
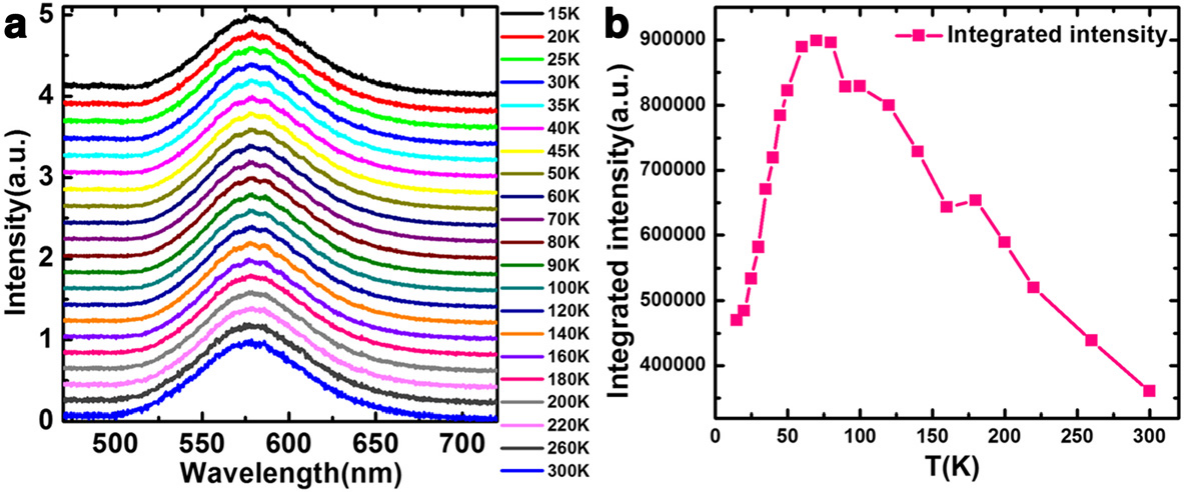
**a** PL spectra of the sample from 15 to 300 K. **b** The PL integrated intensity versus temperature

The PL spectra of the sample over a temperature range from 15 to 300 K are plotted in Fig. 5a. It can be seen that the PL spectra only contain the luminescence of (0001) QW. In principle, the laser spot is so big that it could cover the (0001) plane area and V-shaped pits area. So, the semi-polar QW also can be excited. But the PL spectra do not contain the light-emitting peak of semi-polar QW, which is different from the CL spectra. The reason is tentatively attributed to the fact that the excitation sources are different. The density of carriers excited by high-energy electron beam in CL measurement is much higher than that aroused by a 405-nm laser in PL measurement. In addition, carriers in semi-polar QW may transfer into (0001) QW and/or be captured by dislocations. Therefore, no PL emission from semi-polar QW was observed. In CL experiments, the carrier density is so high that, apart from transferring and capture, there are still enough carriers to combine radiatively.

The PL integrated intensity versus temperature is plotted in Fig. 5b. We can find that it increases significantly up to 70 K and then gradually decreases. The increase of PL integrated intensity up to 70 K further confirms that the localized carriers, caused by composition fluctuations, in semi-polar QW are thermally activated and transfer into (0001) QW to recombine. When further increasing temperature, it begins to decrease due to enhanced nonradiative recombination.

## Conclusions

In summary, we have studied the emission properties of semi-polar InGaN/GaN MQWs in the V-shaped pits. The light-emitting energy level of semi-polar QW is 355.8 meV higher than that of (0001) QW, which prevents carriers in (0001) QW from reaching the dislocations in V-shaped pits. Both the integrated CL intensity and the integrated PL intensity of (0001) QW increase first and then decrease with rising in temperature, demonstrating that the localized carriers by composition fluctuations in semi-polar QW are thermally activated and transfer into (0001) QW with heating up. This leads to the improvement of light emission efficiency of the (0001) InGaN/GaN MQWs. It is clear that the optical properties of (0001) QW can be improved through the formation of semi-polar QW, which is beneficial in fabrication of high-efficiency GaN-based optoelectronic devices.

## Abbreviations

AFM: atomic force microscopy
CCD: charge-coupled device
CL: cathodoluminescence
CW: continuous wave
LED: light-emitting diodes
MOCVD: metal organic chemical vapor deposition
MQW: multi-quantum well
PL: photoluminescence
QCSE: quantum-confined Stark effect
SSL: solid-state semiconductor lighting
TEM: transmission electron microscopy
